# Data on horizontal hydraulic conductivity of fine-grained soils of the former Lake Texcoco (Mexico)

**DOI:** 10.1016/j.dib.2018.06.013

**Published:** 2018-06-14

**Authors:** Norma Patricia López-Acosta, David Francisco Barba-Galdámez, Alejandra Liliana Espinosa-Santiago, Paloma Inés Choque-Mamani

**Affiliations:** Instituto de Ingeniería, UNAM, Mexico

## Abstract

This article contains data of the horizontal hydraulic conductivity of five different fine-grained soils of the former Lake Texcoco. The data were back-calculated from excess pore pressure dissipation measurements collected at 119 locations using piezocone tests (CPTu). The test campaign was part of the geotechnical survey performed for the construction of the New Mexico City International Airport (NAICM). Descriptive statistical parameters of each soil unit are presented and lognormal probability distributions are fitted to describe the natural variability of the horizontal hydraulic conductivity of this site.

## Specifications Table

**Table:** 

Subject area	*Earth science and Civil engineering*
More specific subject area	*Geology and Geotechnical engineering*
Type of data	*Tables and figures*
How data was acquired	*Geotechnical survey using Piezocone tests (CPTu)*
Data format	*Raw and analyzed*
Experimental factors	*–*
Experimental features	*Horizontal hydraulic conductivity of fined-grained soils was indirectly measured using excess pore pressure dissipation records from piezocone tests (CPTu)*
Data source location	*New Mexico City International Airport* (NAICM)*, Texcoco, Mexico. The study area is bounded by latitude 19°28’12’’–19°33’36’’ N and longitude 98°56’24’’–99°01’12’’ W*
Data accessibility	*The data are available within the article*
Related research article	*–*

## Value of the data

• The data allow researchers to analyze spatial variability of the hydraulic conductivity of soil of the former Lake Texcoco.
• The data are necessary for the analyses of geotechnical problems such as uplift pressure in excavations, regional subsidence and the effect of rain infiltrations on the slope stability of lagoons and canals, which have taken on increasing importance for the construction of the New Mexico City International Airport (NAICM) in the area.
• The data are of vital importance in the estimation of the degree of consolidation achieved by the conventional preload technique with prefabricated vertical drains implemented for the construction of the NAICM runways.
• The data and their statistical parameters can be used in the design of pumping systems and soft ground improvement methods for the airport structures under construction.
• The data serve as a reference to estimate the permeability magnitude of Mexico City lacustrine soils when results of field or laboratory test are not available. Additionally, the provided data contain permeabilities of some seams interspersed in the clayey formations, not commonly included in the site subsoil information.

## 1 Data

The dataset of this article provides information about the horizontal hydraulic conductivity of five different fine-grained soils of the former Lake Texcoco. Table 1 shows a brief description of the study area stratigraphy. The horizontal hydraulic conductivity values given in Table 2 were estimated from excess pore pressure dissipation measurements. Table 3 presents the main statistical parameters of each soil unit. Lognormal probability distributions were fitted to describe the variability of the horizontal hydraulic conductivity. Goodness-of-fit tests were performed to evaluate lognormal distribution hypotheses.

**Table 1:** Stratigraphic description of the study area.

**Soil unit**	**USCS classification**	**Description**	**Thickness (m)**	**Unit weight,*****γ*****(kN/m**^**3**^**)**
Surface Crust (SC)	CH	Light brown fat clay of soft consistency	1	15
Upper Clay Formation (UCF)	CH	Very soft and green-gray fat clay interspersed with relatively thin seams of volcanic ash and sandy silt	21–34	12
Hard Layer (HL)	ML	Hard green-gray sandy silt	2	16
Lower Clay Formation (LCF)	CH	Green-brown clay interspersed with gray fat clay	5–12	13
Deep Deposits (DD)	–	Heterogeneous formation of clay, silt and sand	8–10	18
Deep Clay Formation (DCF)	CH	Soft to very firm green-gray clay	9–11	15
Deep Stratified Formation (DSF)	–	Clayey, silty and sandy soils stratifications	16–19	17

**Table 2:** Horizontal hydraulic conductivity of fine-grained soils.

**ID**	**Latitude**	**Longitude**	**Depth (m)**	**Soil unit**	**Horizontal hydraulic conductivity,*****k*****(m/s)**
CPTu-01	19°33׳05.48׳׳N	98°59׳55.40׳׳W	33.14	DD	1.27x10^-^^8^
CPTu-02	19°33׳05.19׳׳N	98°59׳48.50׳׳W	18.62	UCF (S)	1.35x10^-^^8^
			28.84	LCF	3.42x10^-8^
CPTu-03	19°33׳02.58׳׳N	98°59׳09.48׳׳W	25.50	LCF	3.63x10^-9^
CPTu-04	19°33׳00.53׳׳N	98°59׳29.73׳׳W	25.06	LCF	1.47x10^-9^
			34.94	DD	9.20x10^-10^
			39.74	DD	7.23x10^-10^
CPTu-05	19°32׳56.73׳׳N	98°59׳58.01׳׳W	45.42	DCF	2.05x10^-9^
CPTu-06	19°32׳54.86׳׳N	98°58׳04.72׳׳W	14.36	UCF (S)	2.15x10^-8^
			24.58	LCF	6.39x10^-9^
CPTu-07	19°32׳54.00׳׳N	98°59׳52.28׳׳W	33.85	DD	1.02x10^-8^
CPTu-08	19°32׳50.77׳׳N	98°59׳49.33׳׳W	19.02	LCF	1.96x10^-8^
			34.48	DD	5.96x10^-9^
			41.56	DD	2.25x10^-9^
CPTu-09	19°32׳45.24׳׳N	98°59׳58.87׳׳W	56.22	DSF	3.77x10^-9^
CPTu-10	19°32׳45.18׳׳N	99°00׳15.58׳׳W	25.32	LCF	8.14x10^-9^
			41.60	DD	2.37x10^-9^
CPTu-11	19°32׳44.01׳׳N	98°59׳16.62׳׳W	34.98	DD	4.61x10^-9^
CPTu-12	19°32׳41.99׳׳N	98°59׳15.32׳׳W	28.96	LCF	5.96x10^-9^
CPTu-13	19°32׳39.81׳׳N	98°59׳59.42׳׳W	19.54	UCF (S)	7.95x10^-8^
CPTu-14	19°32׳36.90׳׳N	98°58׳02.22׳׳W	40.24	DCF	1.44x10^-9^
CPTu-15	19°32׳35.97׳׳N	98°59׳36.53׳׳W	18.74	UCF (S)	2.54x10^-8^
CPTu-16	19°32׳20.41׳׳W	98°58׳03.98׳׳W	43.50	DCF	7.61x10^-10^
			46.20	DSF	9.29x10^-10^
CPTu-17	19°32׳16.81׳׳N	98°59׳37.83׳׳W	21.25	UCF (S)	2.28x10^-8^
			35.47	DD	3.41x10^-8^
			56.57	DSF	1.22x10^-8^
CPTu-18	19°32׳16.06׳׳N	98°59׳51.56׳׳W	35.86	DD	2.74x10^-8^
			59.46	DSF	1.25x10^-8^
CPTu-19	19°32׳15.74׳׳N	98°59׳59.97׳׳W	25.46	LCF	4.23x10^-9^
			27.40	LCF	9.20x10^-9^
			39.16	DD	3.81x10^-9^
			43.34	DD	4.74x10^-9^
			48.84	DCF	1.50x10^-9^
			56.70	DSF	2.29x10^-9^
CPTu-20	19°32׳15.12׳׳N	98°59׳48.98׳׳W	20.16	UCF (S)	2.99x10^-8^
CPTu-21	19°32׳14.34׳׳N	99°00׳08.13׳׳W	35.14	DD	2.71x10^-8^
CPTu-22	19°32׳13.82׳׳N	98°59׳24.28׳׳W	34.76	DD	7.74x10^-10^
			40.68	DD	7.77x10^-10^
			54.00	DSF	7.56x10^-10^
			60.10	DSF	7.07x10^-10^
CPTu-23	19°32׳11.08׳׳N	98°59׳42.53׳׳W	27.52	LCF	1.53x10^-9^
			42.84	DD	2.08x10^-9^
			56.10	DSF	7.13x10^-10^
CPTu-24	19°32׳09.39׳׳N	98°59׳16.59׳׳W	45.61	DCF	5.10x10^-9^
			54.59	DCF	5.37x10^-9^
			58.71	DSF	7.87x10^-9^
CPTu-25	19°32׳07.05׳׳N	99°00׳15.48׳׳W	24.91	LCF	2.35x10^-9^
			45.52	DCF	1.65x10^-9^
CPTu-26	19°32׳00.70׳׳N	98°59׳23.04׳׳W	53.66	DCF	4.32x10^-10^
			57.50	DSF	3.58x10^-9^
			57.84	DSF	5.73x10^-10^
CPTu-27	19°31׳57.26׳׳N	99°00׳04.05׳׳W	20.52	UCF (S)	5.71x10^-8^
CPTu-28	19°31׳56.93׳׳N	99°00׳07.24׳׳W	21.50	UCF (S)	2.46x10^-8^
			47.76	DCF	7.02x10^-10^
CPTu-29	19°31׳51.53׳׳N	98°59׳52.49׳׳W	35.08	LCF (S)	1.06x10^-8^
			35.28	LCF (S)	2.27x10^-8^
			55.63	DCF (S)	2.15x10^-8^
CPTu-30	19°31׳50.98׳׳N	98°59׳38.42׳׳W	58.22	DSF	6.76x10^-9^
CPTu-31	19°31׳50.78׳׳N	99°00׳16.09׳׳W	44.62	DD	4.07x10^-9^
			48.10	DCF	1.74x10^-9^
CPTu-32	19°31׳48.11׳׳N	98°59׳46.27׳׳W	21.06	UCF	7.58x10^-9^
			37.88	DD	6.86x10^-9^
			43.30	DD	6.74x10^-9^
			55.52	DCF	7.42x10^-9^
			56.58	DSF	4.12x10^-9^
			61.90	DSF	6.29x10^-9^
CPTu-33	19°31׳47.63׳׳N	98°59׳25.24׳׳W	26.70	LCF	2.19x10^-9^
CPTu-34	19°31׳38.58׳׳N	99°00׳09.37׳׳W	37.92	DD	1.58x10^-8^
CPTu-35	19°31׳37.90׳׳N	99°00׳02.33׳׳W	46.79	DCF	7.17x10^-9^
CPTu-36	19°31׳37.83׳׳N	98°58׳23.03׳׳W	21.26	UCF	1.38x10^-9^
			33.64	DD	1.22x10^-9^
			35.06	DD	6.68x10^-10^
CPTu-37	19°31׳36.92׳׳N	98°59׳17.79׳׳W	26.88	LCF	2.75x10^-9^
			42.16	DD	5.68x10^-9^
			45.98	DCF	1.88x10^-9^
CPTu-38	19°31׳29.80׳׳N	98°59׳32.34׳׳W	48.44	DD	2.11x10^-8^
			55.68	DCF	6.17x10^-9^
CPTu-39	19°31׳26.64׳׳N	98°59׳47.10׳׳W	23.38	DD	1.09x10^-8^
			58.08	DSF	4.87x10^-9^
CPTu-40	19°31׳26.06׳׳N	98°59׳40.37׳׳W	23.71	UCF (S)	1.48x10^-8^
CPTu-41	19°31׳21.08׳׳N	98°59׳26.24׳׳W	23.90	UCF	2.05x10^-9^
			56.66	DSF	1.68x10^-9^
CPTU-42	19°31׳20.98׳׳N	98°59׳26.24׳׳W	23.68	UCF	4.38x10^-9^
			38.48	DD	9.76x10^-10^
			49.18	DCF	1.76x10^-9^
			58.70	DSF	3.03x10^-9^
CPTu-43	19°31׳20.65׳׳N	98°59׳18.41׳׳W	23.62	UCF	4.67x10^-9^
CPTu-44	19°31׳18.25׳׳N	99°00׳17.26׳׳W	49.94	DCF	1.63x10^-9^
CPTu-45	19°31׳15.35׳׳N	99°00׳04.36׳׳W	23.58	UCF (S)	1.69x10^-8^
CPTu-46	19°31׳07.32׳׳N	98°59׳47.96׳׳W	40.22	DD	1.62x10^-9^
			61.48	DSF	5.13x10^-10^
CPTu-47	19°31׳04.36׳׳N	98°59׳19.00׳׳W	24.00	UCF (S)	1.54x10^-8^
CPTu-48	19°31׳02.18׳׳N	98°59׳30.66׳׳W	44.00	DSF	1.62x10^-9^
CPTu-49	19°31׳02.05׳׳N	99°00׳08.96׳׳W	29.73	LCF (S)	1.62x10^-8^
			34.87	LCF (S)	1.41x10^-8^
			41.89	DD	2.21x10^-9^
			46.07	DD	1.05x10^-8^
			52.27	DCF (S)	1.29x10^-8^
			67.59	DSF	1.07x10^-9^
CPTu-50	19°31׳01.98׳׳N	99°00׳17.84׳׳W	47.76	DD	1.54x10^-8^
CPTu-51	19°30׳59.28׳׳N	99°00׳07.51׳׳W	30.68	LCF (S)	2.66x10^-8^
CPTu-52	19°30׳59.21׳׳N	98°58׳08.21׳׳W	23.56	UCF	4.26x10^-9^
			43.30	DCF	1.72x10^-9^
CPTu-53	19°30׳59.15׳׳N	99°00׳07.07׳׳W	49.69	DCF	4.14x10^-9^
CPTu-54	19°30׳54.86׳׳N	98°59׳27.23׳׳W	42.47	DD	5.60x10^-9^
			59.21	DSF	1.05x10^-8^
CPTu-55	19°30׳53.00׳׳N	99°00׳00.82׳׳W	48.26	DCF	5.18x10^-9^
CPTu-56	19°30׳52.84׳׳N	99°00׳03.36׳׳W	27.84	DD	4.52x10^-9^
CPTu-57	19°30׳52.38׳׳N	98°59׳35.98׳׳W	61.64	DSF	1.08x10^-8^
			63.66	DSF	5.08x10^-9^
CPTu-58	19°30׳52.02׳׳N	98°59׳12.51׳׳W	21.96	UCF	3.97x10^-9^
			28.84	LCF	3.84x10^-9^
			53.76	DSF	1.50x10^-9^
CPTu-59	19°30׳51.73׳׳N	98°59׳12.51׳׳W	24.22	UCF (S)	7.03x10^-8^
			56.68	DSF	5.15x10^-9^
			64.11	DSF	5.64x10^-9^
CPTu-60	19°30׳51.30׳׳N	98°58׳21.84׳׳W	25.00	LCF	3.05x10^-9^
			27.48	LCF	2.99x10^-9^
			29.02	LCF	1.20x10^-8^
			35.96	DD	3.32x10^-9^
			38.17	DD	2.25x10^-9^
			43.53	DD	5.82x10^-9^
			46.64	DCF	7.67x10^-10^
			50.84	DCF	4.08x10^-9^
			54.64	DSF	1.79x10^-9^
			59.48	DSF	6.61x10^-10^
CPTu-61	19°30׳50.03׳׳N	98°58׳11.17׳׳W	37.00	DD	1.65x10^-8^
			52.72	DSF	5.56x10^-9^
CPTu-62	19°30׳48.74׳׳N	98°58׳59.48׳׳W	25.58	LCF (S)	1.39x10^-8^
CPTu-63	19°30׳46.66׳׳N	99°00׳09.81׳׳W	39.26	LCF (S)	2.14x10^-8^
CPTu-64	19°30׳45.71׳׳N	99°00׳18.46׳׳W	27.40	UCF	9.31x10^-9^
			30.58	LCF	4.42x10^-9^
			32.16	LCF (S)	2.84x10^-8^
			49.44	DCF	2.82x10^-9^
			52.80	DCF	4.04x10^-9^
CPTu-65	19°30׳43.05׳׳N	98°59׳48.33׳׳W	24.38	UCF (S)	1.05x10^-8^
			42.34	DD	9.84x10^-10^
			43.96	DD	1.85x10^-9^
			45.30	DD	5.84x10^-10^
			48.32	DCF	1.14x10^-9^
			52.14	DCF	6.20x10^-10^
CPTu-66	19°30׳37.09׳׳N	98°59׳57.56׳׳W	28.17	UCF (S)	1.93x10^-8^
			63.89	DSF	1.54x10^-8^
			71.21	DSF	1.98x10^-8^
CPTu-67	19°30׳37.00׳׳N	98°59׳48.95׳׳W	27.06	UCF	9.15x10^-9^
CPTu-68	19°30׳36.57׳׳N	98°59׳40.24׳׳W	30.36	LCF (S)	1.35x10^-8^
CPTu-69	19°30׳33.51׳׳N	99°00׳11.94׳׳W	41.28	DD	1.00x10^-8^
			65.64	DSF	2.16x10^-8^
CPTu-70	19°30׳31.85׳׳N	98°59׳20.23׳׳W	27.10	HL	1.34x10^-8^
			47.82	DCF	4.03x10^-9^
CPTu-71	19°30׳31.11׳׳N	98°59׳14.47׳׳W	41.84	DD	9.66x10^-10^
			59.60	DSF	8.55x10^-10^
CPTu-72	19°30׳29.70׳׳N	98°57׳52.78׳׳W	53.98	DSF	8.87x10^-10^
CPTu-73	19°30׳29.06׳׳N	98°59׳28.19׳׳W	27.50	UCF (S)	1.32x10^-8^
			40.74	DD	2.42x10^-8^
CPTu-74	19°30׳28.80׳׳N	99°00׳01.54׳׳W	40.28	LCF (S)	4.06x10^-8^
			64.94	DSF	7.77x10^-9^
CPTu-75	19°30׳28.57׳׳N	98°59׳35.06׳׳W	45.42	DD	1.74x10^-8^
			46.12	DD	8.17x10^-9^
			60.30	DSF	3.41x10^-9^
CPTu-76	19°30׳27.03׳׳N	98°58׳04.55׳׳W	50.06	DCF	2.50x10^-9^
			63.90	DSF	3.97x10^-10^
CPTu-77	19°30׳21.15׳׳N	98°59׳58.04׳׳W	72.48	DSF	8.16x10^-9^
CPTu-78	19°30׳20.86׳׳N	98°59׳48.44׳׳W	28.18	UCF (S)	3.38x10^-8^
			33.06	LCF (S)	4.11x10^-8^
			41.66	DD	1.03x10^-8^
			44.28	DD	1.57x10^-8^
			64.62	DSF	2.17x10^-9^
CPTu-79	19°30׳20.40׳׳N	98°59׳41.23׳׳W	27.74	UCF	5.16x10^-9^
			28.43	UCF	4.41x10^-9^
			46.05	DD	1.17x10^-8^
			72.04	DSF	5.75x10^-9^
CPTu-81	19°30׳17.80׳׳N	99°00׳06.28׳׳W	66.24	DSF	4.17x10^-8^
CPTu-82	19°30׳17.35׳׳N	98°59׳59.93׳׳W	29.46	UCF	8.83x10^-9^
CPTu-83	19°30׳16.08׳׳N	98°59׳27.06׳׳W	29.98	UCF	1.32x10^-8^
			44.02	DD	7.98x10^-9^
			60.52	DCF	7.95x10^-10^
CPTu-84	19°30׳15.65׳׳N	98°59׳20.03׳׳W	29.98	LCF	5.48x10^-9^
			51.46	DCF	2.61x10^-9^
CPTu-85	19°30׳13.50׳׳N	98°58׳58.14׳׳W	28.98	UCF (S)	2.28x10^-8^
CPTu-86	19°30׳13.15׳׳N	99°00׳19.73׳׳W	48.94	DD	1.23x10^-8^
			55.04	DCF	1.64x10^-9^
CPTu-87	19°30׳12.73׳׳N	98°59׳51.25׳׳W	44.00	DD	8.92x10^-9^
CPTu-88	19°30׳04.81׳׳N	98°57׳57.24׳׳W	30.60	LCF	1.63x10^-8^
			55.48	DSF	2.07x10^-9^
CPTu-89	19°30׳04.72׳׳N	98°59׳50.02׳׳W	31.04	UCF	7.10x10^-9^
			52.96	DCF	4.63x10^-9^
			69.76	DSF	5.11x10^-9^
CPTu-90	19°30׳04.40׳׳N	98°59׳58.25׳׳W	36.85	LCF	4.80x10^-9^
			45.67	DD	1.29x10^-8^
			50.11	DD	1.18x10^-8^
			66.83	DSF	6.72x10^-9^
CPTu-91	19°30׳04.04׳׳N	98°59׳41.78׳׳W	31.44	LCF (S)	2.51x10^-8^
			65.58	DSF	5.04x10^-9^
CPTu-92	19°30׳03.40׳׳N	98°57׳31.58׳׳W	46.68	DCF	8.90x10^-10^
CPTu-93	19°30׳03.02׳׳N	98°57׳45.89׳׳W	38.19	DD	9.09x10^-9^
			50.97	DCF	2.33x10^-9^
CPTu-94	19°29׳29.55׳׳N	98°59׳35.95׳׳W	43.16	LCF	9.16x10^-9^
			44.76	DD	1.26x10^-8^
			63.37	DSF	2.90x10^-9^
CPTu-95	19°29׳57.01׳׳N	99°00׳19.18׳׳W	55.94	DCF	2.84x10^-9^
CPTu-96	19°29׳56.68׳׳N	98°58׳19.03׳׳W	56.60	DSF	2.43x10^-10^
CPTu-97	19°29׳56.41׳׳N	98°57׳31.21׳׳W	25.08	UCF (S)	3.56x10^-8^
CPTu-98	19°29׳56.31׳׳N	98°57׳24.93׳׳W	51.38	DSF	2.41x10^-9^
CPTu-99	19°29׳55.16׳׳N	99°00׳22.03׳׳W	55.52	DCF	2.87x10^-9^
CPTu-100	19°29׳54.97׳׳N	98°57׳09.35׳׳W	22.84	UCF (S)	4.98x10^-8^
CPTu-101	19°29׳53.14׳׳N	98°59׳19.83׳׳W	29.98	UCF	8.33x10^-9^
CPTu-102	19°29׳51.74׳׳N	99°00׳01.17׳׳W	36.16	LCF	1.29x10^-7^
			52.46	DCF	5.89x10^-9^
			56.88	DCF	2.45x10^-9^
CPTu-103	19°29׳50.44׳׳N	99°00׳47.07׳׳W	34.52	LCF	2.71x10^-9^
CPTu-104	19°29׳50.08׳׳N	99°00׳39.80׳׳W	33.02	LCF	6.70x10^-9^
			39.96	LCF	6.22x10^-9^
			41.68	LCF	8.09x10^-9^
			50.36	DD	5.99x10^-9^
			53.24	DCF	1.31x10^-9^
			54.64	DCF	8.18x10^-9^
CPTu-105	19°29׳49.66׳N	98°59׳05.28׳׳W	63.48	DSF	2.27x10^-9^
CPTu-106	19°29׳48.62׳׳N	98°59׳22.23׳׳W	53.32	DCF	1.49x10^-9^
CPTu-107	19°29׳48.16׳׳N	98°59׳26.17׳׳W	30.54	UCF	2.40x10^-8^
CPTu-108	19°29׳47.90׳׳N	98°59׳30.67׳׳W	54.68	DCF	4.65x10^-9^
CPTu-109	19°29׳43.74׳׳N	98°59׳30.94׳׳W	20.00	UCF	2.00x10^-9^
			40.00	LCF	5.60x10^-10^
CPTu-110	19°29׳43.09׳׳N	98°59׳31.66׳׳W	20.00	UCF	2.00x10^-9^
			40.00	LCF	6.50x10^-10^
CPTu-111	19°29׳37.82׳׳׳N	98°56׳51.79׳׳W	39.78	DD	1.58x10^-8^
CPTu-112	19°29׳36.06׳׳N	98°59׳31.49׳׳W	5.50	UCF	8.70x10^-9^
			8.34	UCF	5.69x10^-9^
			16.30	UCF	6.29x10^-9^
			19.00	UCF (S)	1.91x10^-9^
			24.00	UCF	1.81x10^-9^
			28.80	UCF	9.41x10^-10^
CPTu-113	19°29׳35.28׳׳N	98°59׳32.83׳׳W	5.51	UCF	5.21x10^-9^
			8.30	UCF	9.99x10^-9^
			16.30	UCF	1.71x10^-9^
			19.01	UCF (S)	2.34x10^-9^
			24.39	UCF (S)	1.16x10^-9^
			28.81	UCF	8.31x10^-10^
CPTu-114	19°29׳35.21׳׳N	98°59׳31.53׳׳W	5.50	UCF	8.70x10^-9^
			8.30	UCF	6.66x10^-9^
			10.26	UCF (S)	2.45x10^-8^
			16.30	UCF	5.98x10^-9^
			20.50	UCF	4.35x10^-9^
			23.32	UCF (S)	1.95x10^-8^
			24.00	UCF	2.71x10^-9^
			25.10	UCF (S)	1.62x10^-8^
			28.80	UCF	4.21x10^-10^
CPTu-115	19°29׳35.18׳׳N	98°59׳30.26׳׳W	5.50	UCF	6.52x10^-9^
			8.30	UCF	3.99x10^-9^
			16.30	UCF	2.99x10^-9^
			19.00	UCF (S)	1.11x10^-8^
			22.84	UCF	1.24x10^-9^
			24.00	UCF	1.55x10^-9^
			24.78	UCF	2.50x10^-9^
			28.00	UCF	1.02x10^-9^
CPTu-116	19°29׳34.27׳׳N	98°59׳31.56׳׳W	4.50	UCF	3.26x10^-9^
			9.00	UCF	9.43x10^-9^
			16.54	UCF	5.37x10^-9^
			20.36	UCF	5.24x10^-9^
			28.08	UCF	2.35x10^-9^
			29.08	UCF	2.50x10^-9^
			30.60	HL	1.76x10^-8^
CPTu-117	19°29׳30.45׳׳N	98°57׳39.28׳׳W	48.88	DCF	2.09x10^-9^
CPTu-118	19°29׳27.34׳׳N	99°00׳21.06׳׳W	34.02	LCF	1.86x10^-8^
CPTu-119	19°29׳15.09׳׳N	98°57׳49.30׳׳W	27.14	UCF (S)	1.50x10^-8^

Note: (S) = seam.

**Table 3:** Summary of horizontal hydraulic conductivity statistics of fined-grained soil layers.

**Soil unit**	***n***	**Minimum (m/s)**	**Maximum (m/s)**	***m***_***k(A)***_**(m/s)**	***s***_***k(A)***_**(m/s)**	**CV**	***g***_***1***_	***g***_***2***_	***m***_***k(G)***_**(m/s)**	***s***_***k(G)***_**(m/s)**
UCF	46	4.21×10^−10^	2.40×10^−8^	5.21×10^−9^	4.14×10^−9^	0.80	2.24	11.31	3.88×10^−9^	2.28
LCF	30	5.60×10^−10^	3.42×10^−8^	7.19×10^−9^	7.09×10^−9^	0.99	2.31	9.54	4.84×10^−9^	2.56
DD	57	5.84×10^−10^	3.41×10^−8^	8.50×10^−9^	7.66×10^−9^	0.90	1.31	4.65	5.19×10^−9^	3.05
DCF	45	4.32×10^−10^	8.18×10^−9^	2.99×10^−9^	2.01×10^−9^	0.67	0.87	2.90	2.33×10^−9^	2.09
DSF	50	2.43×10^−10^	2.16×10^−8^	4.95×10^−9^	4.93×10^−9^	1.00	1.65	5.80	2.93×10^−9^	3.07

Note: *n* = sample size, *m*_*k(A)*_ = arithmetic mean, *s*_*k*(A)_ = arithmetic standard deviation, CV = coefficient of variation, *g*_*1*_ = skewness, *g*_*2*_ = kurtosis, *m*_*k(G)*_ = geometric mean, *s*_*k(G)*_ = geometric standard deviation.

## 2 Experimental design, materials, and methods

### 2.1 Description of the study area

**Fig. 1:**
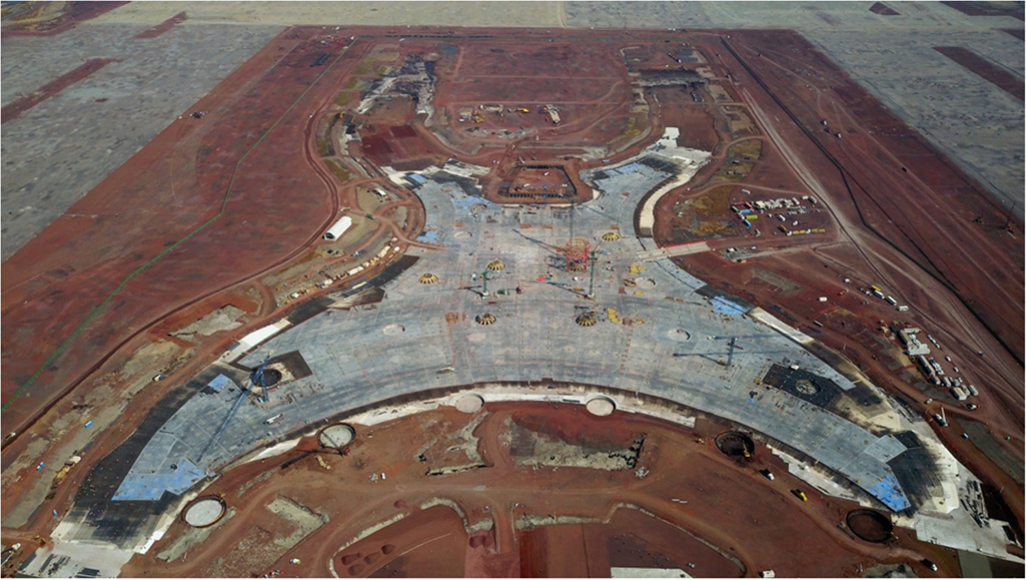
Progress in the foundation slab of the Terminal Building of the NAICM to February 26, 2018 (photo courtesy of M.J. Mendoza-López).

The study area is located at the lowest portion of the Basin of Mexico, formerly occupied by Lake Texcoco (State of Mexico, Mexico). The basin of Mexico is a compound graben surrounded by tertiary-quaternary mountain ranges. The basin was originally open until 700,000 years ago when andesitic lava flows blocked its drainage resulting in the development of an extensive system of lakes [1]. The former Lake Texcoco was the largest and shallowest lake in the Basin. Groundwater in the area is characterized by its high salinity (up to 10 g/l) and alkalinity (above 10 meq/l). The topography is plain and vegetation is dominated by halophytic plants [2], [3]. Texcoco soils consists of thick layers of soft clay of lacustrine origin with microfossils (benthic and epiphytic diatoms) interlayered by volcanic sediments [2]. In recent years, this 33.6-square-kilometer zone has been subjected to rapid urbanization because of the construction of the New Mexico City International Airport (NAICM, Fig. 1). This process requires the analysis, design and construction of several geotechnical works for which the knowledge of hydraulic properties of the soils is vital. Namely, the permeability of the seams interspersed in the clayey formations is an important data to evaluate stability against uplift pressure in excavations, design of pumping systems and improvement of soft ground, among others.

The site stratigraphy corresponds to typical Lacustrine Zone according to Mexico City Geotechnical Zoning [4]. As seen in Table 1, the main soil units are: a) Surface Crust (SC) formed by fat clay sediments with the presence of cracks; b) Upper Clay Formation (UCF), a thick layer of highly compressible lacustrine clay interspersed with seams of volcanic origin; c) Hard Layer (HL) composed of a series of thin layers of sand and silt with variable cementation; d) Lower Clay Formation (LCF) of the same origin of the UCF, it differs by its lower water content and compressibility; e) Deep Deposits (DD) formed by silts and sand interspersed with hard clays; f) Deep Clay Formation (DCF), a third clay layer with similar characteristics to the others; and g) Deep Stratified Formation (DSF) composed of stratified deposits of clay, sand and silty sand [5], [6]. In the site the groundwater table level (GTL) is 1.0 m depth (variable depending on the rainy and dry seasons).

### 2.2 Estimation of horizontal hydraulic conductivity

**Fig. 2:**
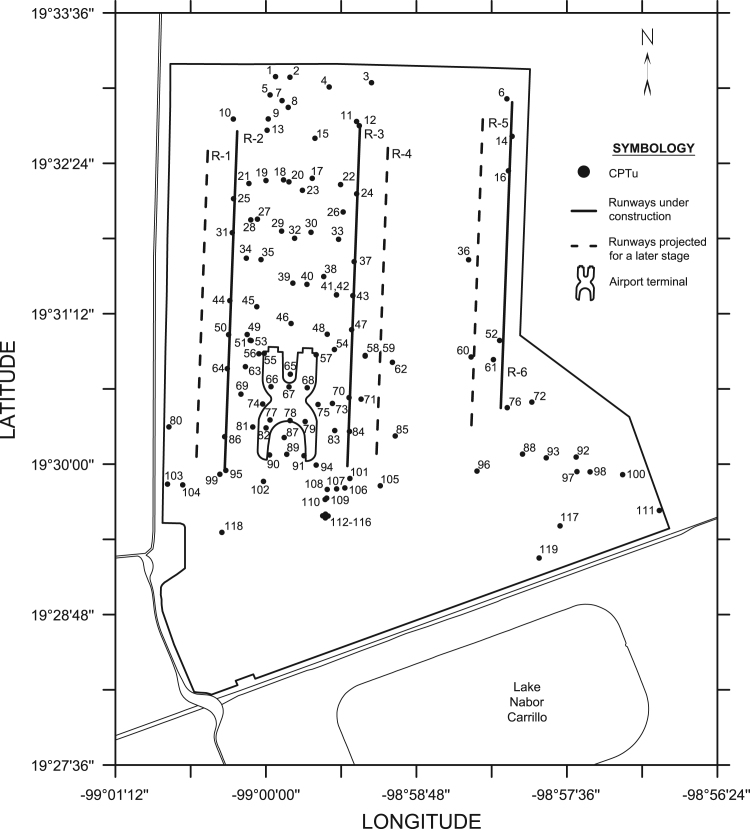
Former Lake Texcoco base map and CPTu locations.

Piezocone tests (CPTu) were performed at 119 locations across the study area (Fig. 2). CPTu is an *in-situ* testing method used to estimate geotechnical properties of soils. The test consists of driving an instrumented steel probe into the ground at a constant speed (2 cm/s) to obtain a continuous record of resistance to penetration and pore water pressure [7]. The piezocone features allow to measure the dissipation of excess pore water pressure caused by cone penetration. These data can be employed to back-calculate the horizontal hydraulic conductivity (*k*) of fine-grained soils. Several interpretation theories have been proposed to obtain this parameter [8], [9], [10], [11], [12]. In the present article, the empirical method of Baligh and Levadoux [9] is employed:

(1) $$k=\frac{\gamma_{w}}{2.3p_{\mathrm{vo}}^{\prime }\;}\cdot \mathrm{RR}\cdot c_{h}$$

where *p’*_*vo*_ is the initial vertical effective stress, *γ*_*w*_ is the unit weight of water, *RR* is the recompression ratio controlling dissipation around the piezocone and *c*_*h*_ is the horizontal coefficient of consolidation defined by:

(2) $${\text{c}}_{\text{h}\;}\text{=}\;\frac{R^{2}T}{t}$$

where *R* is the radius of the cone shaft, *t* is the measured time to reach a certain degree of consolidation and *T* is a time factor.

### 2.3 Statistical data processing

Table 3 presents a summary of the main statistical parameters of the clayey soil units (UCF, LCF, DD, DCF and DSF). The data of the Hard Layer (HL) and the silty seams (S) were not analyzed because of the limited number of measurements.

Hydraulic conductivity is a soil property that exhibits a significant natural variability. Several authors have suggested that this property can be described satisfactorily with the two-parameter lognormal distribution [13], [14], [15], [16], [17], [18]. In this article, the adjustment to both natural (ln *k*) and decimal logarithms (log *k*) are presented. The former are more widely used in literature, however the latter are easier to relate to the original value of the hydraulic conductivity [19]. The model estimators were calculated according to the Maximum Likelihood Method [20]:

(3) $$\mu_{\log_{\text{a}}\text{k}}\text{=}\frac{\text{1}}{\text{n}}\overset{\text{n}}{\underset{i=1}{\sum }}\log_{\text{a}}{\text{k}}_{\text{i}}$$

(4) $$\sigma_{\log_{\text{a}}\text{k}\;}\text{=}\;\frac{\text{1}}{\text{n}\text{-1}}\overset{\text{n}}{\underset{i=1}{\sum }}{\left(\log_{\text{a}}{\text{k}}_{\text{i}}\text{-}\mu_{\log_{\text{a}}\text{k}}\right)}^{\text{2}}$$

where $\mu_{{\text{log}}_{\text{a}}k}$ is the mean of the logarithm base *a* of *k*, $\sigma_{{\text{log}}_{a}k}$ is the standard deviation of the logarithm base *a* of *k*, and *n* is the sample size.

**Fig. 3:**
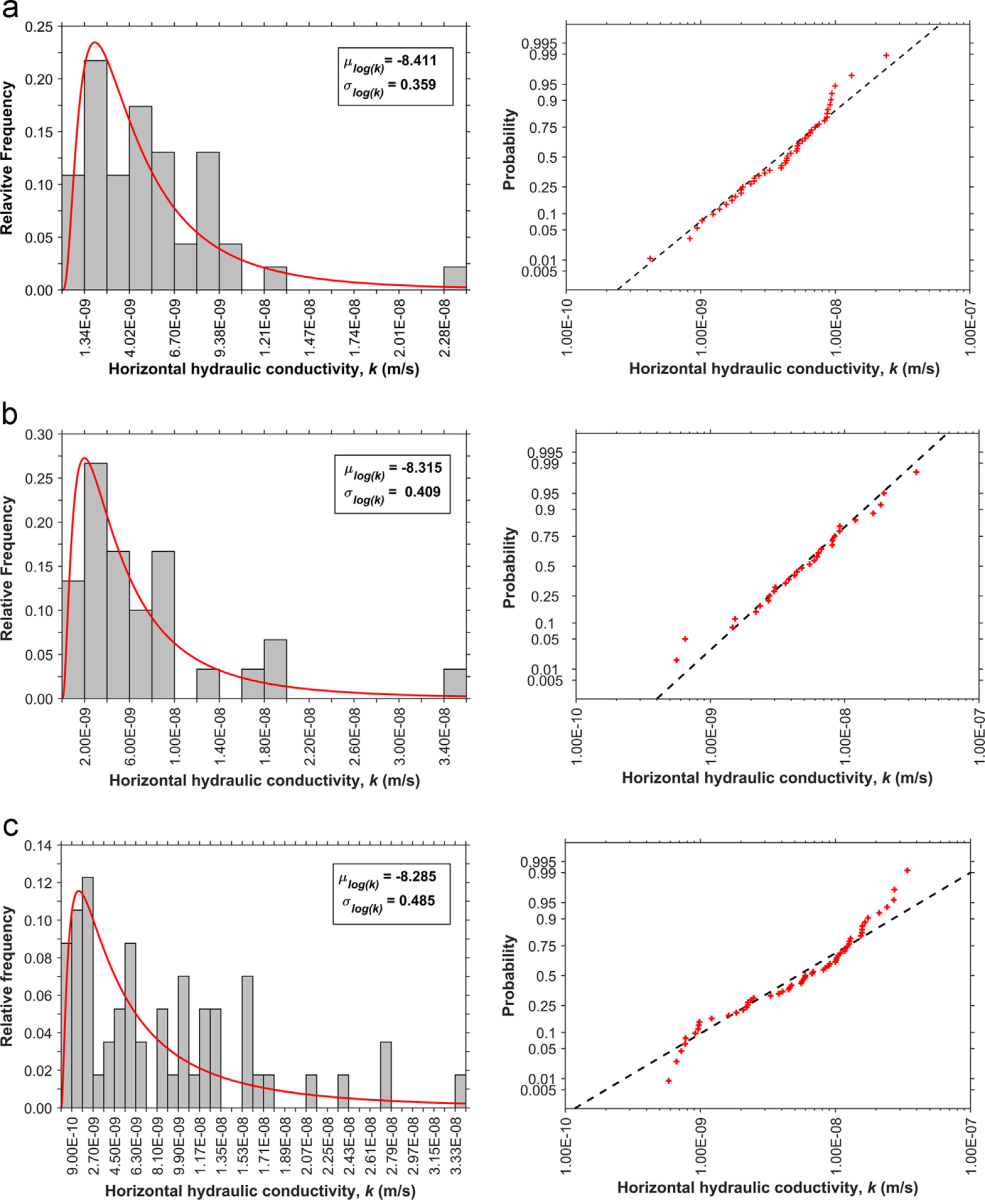
Histograms and probability plots for (a) UCF, (b) LCF, (c) DD.

**Fig. 4:**
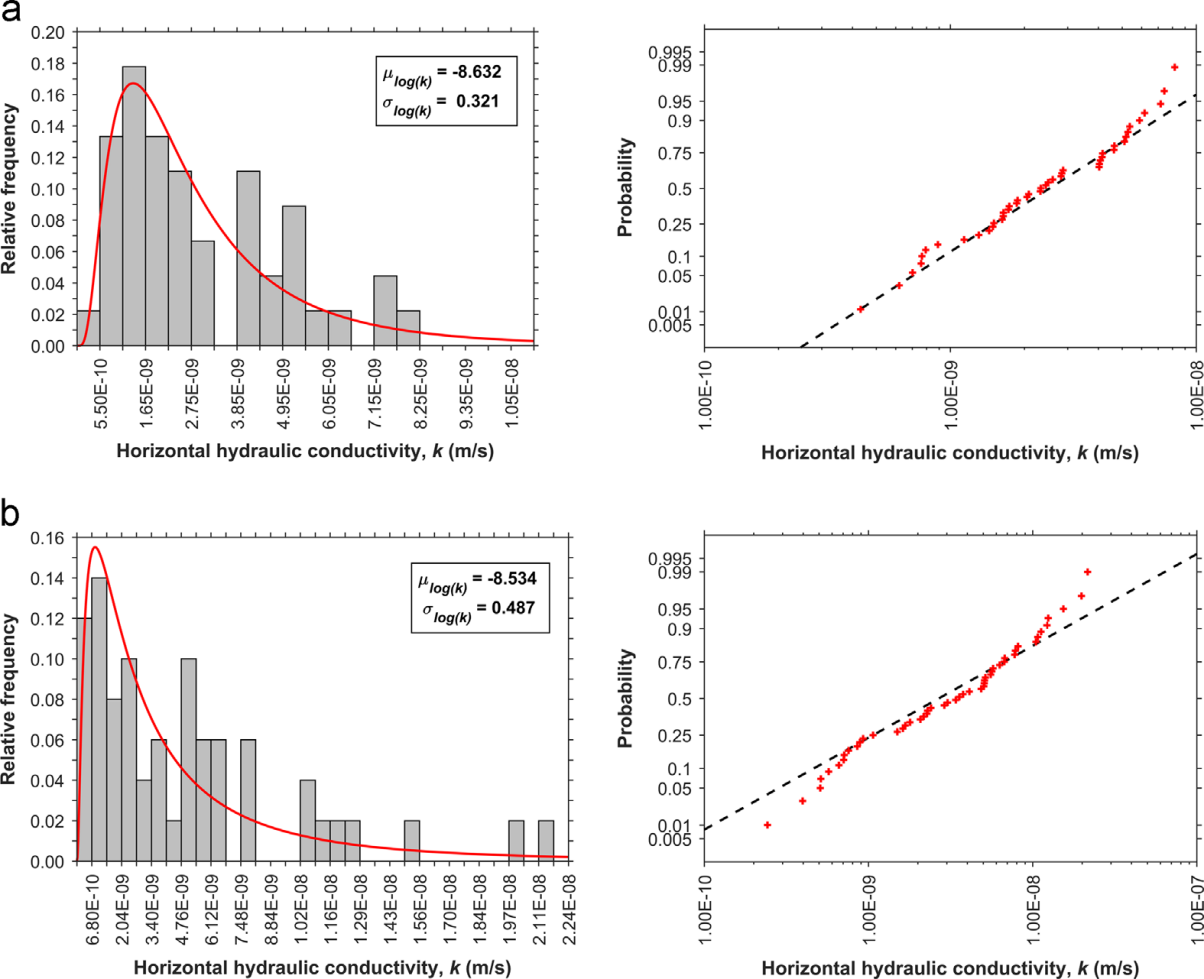
Histograms and probability plots for (a) DCF, (b) DSF.

Fig. 3, Fig. 4 show the relative frequency histograms and the cumulative distributions on lognormal probability plots for the horizontal hydraulic conductivity of each soil unit. Kolmogorov–Smirnov tests (K–S test) at a 5% level of significance were conducted to verify the goodness-of-fit of the theoretical probability distributions [21]. The null hypothesis implies that the sample is drawn from a lognormal probability density. Table 4 presents the estimated model parameters and the results of the goodness-of-fit tests.

**Table 4:** Summary of lognormal probability distribution parameters and results of the goodness-of-fit tests.

**Soil unit**	**Fitted parameters**				**K–S test**		
	***μ***_**ln(*****k*****)**_	***σ***_**ln(*****k*****)**_	***μ***_**log(*****k*****)**_	***σ***_**log(*****k*****)**_	**Statistic**	***P*-value**	**Test result**
UCF	− 19.3669	0.8258	− 8.411	0.359	0.119	0.494	NR
LCF	− 19.1460	0.9418	− 8.315	0.409	0.138	0.574	NR
DD	− 19.0761	1.1162	− 8.285	0.485	0.114	0.422	NR
DCF	− 19.8760	0.7390	− 8.632	0.321	0.126	0.435	NR
DSF	− 19.6499	1.1220	− 8.534	0.487	0.115	0.489	NR

Note: *μ*_*ln(k)*_ = mean of the natural logarithm of the data, *σ*_*ln(k)*_ = standard deviation of the natural logarithm of the data, *μ*_*log(k)*_ = mean of the decimal logarithm of the data, *σ*_*log(k)*_ = standard deviation of the decimal logarithm of the data, *NR* = non-rejection of the null hypothesis.
