# Supplementary material for: Data on horizontal hydraulic conductivity of fine-grained soils of the former Lake Texcoco (Mexico)
Source: Data Brief. 2018 Jun 14;19:1670–82. doi: 10.1016/j.dib.2018.06.013 (PMC6141130; doi:10.1016/j.dib.2018.06.013)
Supplement: Supplementary file 1 — Supplementary material [file mmc1.pdf]

Conflict of Interest Form

Mexico City
May 28th, 2018

Author Declaration

We wish to confirm that there are no known conflicts of interest associated with this publication and we confirm that we have mentioned all organizations that funded our research in the Acknowledgements section of our submission.

We confirm that the manuscript has been read and approved by all named authors and that there are no other persons who satisfied the criteria for authorship but are not listed. We further confirm that the order of authors listed in the manuscript has been approved by all of us.

We confirm that we have given due consideration to the protection of intellectual property associated with this work and that there are no impediments to publication, including the timing of publication, with respect to intellectual property. In so doing we confirm that we have followed the regulations of our institutions concerning intellectual property.

We understand that the Corresponding Author is the sole contact for the Editorial process (including Editorial Manager and direct communications with the office). She is responsible for communicating with the other authors about progress, submissions of revisions and final approval of proofs. We confirm that we have provided a current, correct email address which is accessible by the Corresponding Author and which has been configured to accept email from nlopeza@iingen.unam.mx

Signed by all authors as follows:

**Author's full name**	**Signature**	**Date**
Dr. Norma Patricia LÓPEZ-ACOSTA (corresponding author)		May 28th, 2018
M.Eng. David Francisco BARBA-GALDÁMEZ		May 28th, 2018
M.Eng. Alejandra Liliana ESPINOSA-SANTIAGO		May 28th, 2018
C.Eng. Paloma Inés CHOQUE-MAMANI		May 28th 2018
